# Prevalence and clinical features of adverse food reactions in Portuguese children

**DOI:** 10.1186/s13223-017-0212-y

**Published:** 2017-09-06

**Authors:** Arminda Jorge, Elisa Soares, Emanuel Sarinho, Felix Lorente, Jorge Gama, Luís Taborda-Barata

**Affiliations:** 1 CICS-Health Sciences Research Centre, University of Beira Interior, Avenida Infante D. Henrique, Covilhã, 6200-506 Portugal; 2 Department of Paediatrics, Cova da Beira Hospital, Covilhã, Portugal; 3 UFPE Allergy & Clinical Immunology Research Centre, Pernambuco Federal University, Recife, Brazil; 4 Department of Paediatrics, Salamanca University Hospital, Salamanca, Spain; 5 Department of Mathematics, Faculty of Sciences, University of Beira Interior, Covilhã, Portugal; 6 Department of Allergy & Clinical Immunology, Cova da Beira Hospital, Covilhã, Portugal

**Keywords:** Adverse food reaction, Children, Food allergy, Prevalence

## Abstract

**Background:**

The prevalence of adverse food reactions (AFR) has been increasing in the western world. Clinical manifestations are diversified and it may not be possible to clinically discriminate between IgE and non-IgE mediated AFR. In Portugal, the prevalence of AFR and food allergies in children is not known. Thus, the objectives of this study were to determine the prevalence of AFR in central Portugal.

**Methods:**

Point prevalence study in 3–11 year-old schoolchildren from Central Portugal. Food-related questionnaires, skin prick tests (SPT) with foods and determination of food-specific IgE levels were performed.

**Results:**

Of 4045 schoolchildren, 2474 (61.2%) accepted to be included in the study. Global prevalence of AFR was 7.1% (95% CI 6.2–8.1), based upon the initial questionnaire, 4.6% (95% CI 3.9–5.5), based upon a confirmatory questionnaire and the prevalence of probable food allergy (IgE-associated AFR: positive history + positive SPT and/or positive specific IgE) was 1.4% (95% CI 0.9–1.9). Most frequently implicated foods were fresh fruits, fish and egg. A first episode at an earlier age, mucocutaneous and anaphylactic reactions were more frequent in IgE-associated AFR.

**Conclusions:**

The prevalence of probable food allergy in 3–11 year old Portuguese children from central Portugal is low and parents over-report its frequency. Most frequently implicated foods were fresh fruit and fish. Immediate type, polysymptomatic, and more severe reactions may commence at an earlier age and be more frequent in IgE-associated than in non-IgE associated reactions.

**Electronic supplementary material:**

The online version of this article (doi:10.1186/s13223-017-0212-y) contains supplementary material, which is available to authorized users.

## Background

The prevalence of adverse reactions to foods (AFR) has been increasing, particularly in the first years of life [1–3]. According to a recent metanalysis, the prevalence of self-reported food allergies varies between 3 and 35%, depending upon the age group, the geographical area and the methodology used [4]. This broad range of values may have to do with different methodological approaches which were used in the various studies; furthermore, in some of the reports the sample of involved only children followed up in specialty clinics whereas in other studies the values were obtained in the general population of children; finally, in some cases, these self-reported values were backed up by oral provocation studies whereas in other reports, only questionnaire-based results were used. Nevertheless, there is a scarcity of studies carried out in the general population of children.

The diagnosis of food allergies in children cannot be made exclusively on the basis of reported symptoms since although parents believe that their children are allergic to foods, confirmation only takes place in 10–50% of the reported cases [5, 6]. For instance, a review of five studies of food allergies in unselected pediatric populations under 10 years of age concluded that the prevalence of these allergies was higher when it was based upon self-reports than when it was supported by additional tests, which suggests that confirmatory allergy tests must be performed [2].

Most frequently reported foods in association with allergies in children are cow’s milk, egg, peanut and wheat, and clinical manifestations range from localized cutaneous reactions to life-threatening anaphylaxis [6–8]. The most efficient therapeutic option for food allergies is food eviction [6, 8]. It is, therefore, important to distinguish between situations of true IgE-associated food allergies and other situations that may involve intolerance to food, other forms of non-IgE-mediated food allergy and even common paediatric gastroenterological situations and this is where allergy tests and, when necessary, food challenges are required [9]. Independently of oral provocation tests remaining the “gold standard”, skin prick tests (SPT) and determination of levels of specific IgE should be performed if the clinical history strongly suggests food allergy and there is a clear suspicion of the implicated specific foods [6].

In Portugal, studies on the prevalence of food allergies are scarce [10] with a single study which analysed children attending an allergy outpatient clinic finding a prevalence of 8.7% [11]. However, in contrast to the aims of our study, that report was not carried out in the general population and include children and adolescents up to 18 years of age. Thus, the objective of the present study was to determine the prevalence of IgE-associated food allergy in children selected from the general population of Central Portugal, and to characterize it in terms of implicated foods and clinical manifestations, in comparison with cases of non-IgE associated AFR.

## Methods

### Study design

Population-based, cross-sectional study, carried out in a 2 year-long period (2012–2013). All 3–11 year old children registered at the existing 53 pre-schools and 69 primary schools in the region of Cova da Beira were recruited for the study. An initial, screening questionnaire about AFR (Q1), containing questions about sociodemographic aspects, the previous occurrence and identification of food associated with the adverse reaction, was filled out by parents. When at least one food was identified as a potential trigger for a previous AFR, a second, previously tested, analysed for cross cultural validation [12] and more comprehensive questionnaire (Q2) was applied by the researchers to fully characterize reactions (Additional file 1). When Q1 and Q2 were both positive, SPT were performed and suspected food-specific IgE levels were determined. The most severe reaction induced by each food was used to characterize the pattern of the reaction [13, 14].

### Diagnosis

Probable food allergy (IgE-associated AFR) was considered in children with a clinical history that suggested previous AFR (positive Q1 and Q2) and who also had positive in vivo (food-specific SPT) and/or in vitro (food-specific IgE levels) allergy studies. A non IgE-associated AFR (non-IgE-AFR) was considered in children with a clinical history suggesting AFR (positive Q1 and Q2) but who had negative in vivo and in vitro food-specific allergy tests [6].

In vivo studies included SPT (LETI Laboratories, Spain) and/or skin prick-prick tests (SPPT) with the suspected food. Tests were carried out in duplicate on the volar aspect of the forearms. A drop of each commercial extract was placed upon the skin and each drop was pricked through using a plastic Stallerpoint (Stallergenes, France). The mean weal diameter was recorded. Weals with a mean diameter at least 3 mm greater than that of the negative control were regarded as positive. SPPT tests used the same methodology but were only performed using fresh fruits. A 25-gauge needle was inserted into fruits and the juice obtained was placed upon the skin and pricked through with Stallerpoints.

SPT with aeroallergen extracts were also performed using the European standard battery of aeroallergens [15].

In vitro tests consisted of the determination of serum levels of food-specific IgE for each suspected food, using a fluorometric method (Unicap 100 Phadia Diagnosis, Thermo Scientific^®^, Uppsala, Sweden) and were regarded as positive when levels were equal to or greater than 0.35 KUA/L. A similar analysis was also performed for screening of sensitisation to aeroallergens (Phadiatop; Phadia Diagnosis; Thermo Scientific^®^, Uppsala, Sweden).

### Statistical analysis

Data were analysed using the Software Package for Social Sciences (SPSS) version 19.0^®^. Analysis of normality of distribution of variables was performed using the one sample Kolmogorov–Smirnov test. Descriptive analysis was used for the characterization of the sample. Chi Square test or Fischer’s Exact Test were used in the case of nominal variables. Comparative analysis of quantitative variables was carried out using Student’s t test or Mann–Whitney U test depending on distribution of variables. For each categorical variable, the “normal” situation was defined as the reference category and odds ratios values were estimated for the other categories against the reference one. A *p* value of less than 0.05 was regarded as significant with all statistical tests.

### Ethics, consent and permissions

This study was approved by the Ethics Committees of the Faculty of Health Sciences, University of Beira Interior and the Ethics Committee of Cova da Beira Hospital Centre. A written informed consent was signed by parents. Questionnaires applied at schools were approved by the general board for curricular innovation and development.

## Results

### Characterization of the population

Of the 4045 children from the target population, the initial questionnaire (Q1) was filled in by the parents of 2474 children (61.2% reply rate) whose mean age was 7.1 ± 1.9 years and 49.9% were males. Socio-demographic features of studied children are shown in Table 1.

**Table 1:** Socio-demographic features of studied children

Parameters	AFR	IgE AFR	Non IgE AFR	Odds ratio IgE AFR*/*Non IgE AFR	*p* value	Target cohort	Q1^+^	Q2^+^
	(n = 109)	(n = 34)	(n = 75)	(95% CI)		(n = 2474)	(n = 176)	(n = 115)
Sex (%)								
F	49.5	23.5	61.3	1	<0.001*	50.1	51.2^a^	48.7^b^
M	50.5	76.5	38.7	5.155 (2.057, 12.918)		49.9	48.8^a^	51.3^b^
Age (years) (mean ± SD)	7.00 ± 1.82	6.65 ± 1.56	7.16 ± 1.91	–	0.173**	7.1 ± 1.9	7.1 ± 1.85	7.0 ± 1.82
Atopy (%)								
No	47.6	9.4	64.4	1	<0.001*	–	–	–
Yes	52.4	90.6	35.6	17.474 (4.851, 62.948)		–	–	–
History of other morbidities (%)								
No	56.0	61.8	53.3	1	0.411*	–	–	–
Yes	44.0	38.2	46.7	0.707 (0.309, 1.618)		–	–	–
Responding parent (%)								
Father	12.0	11.8	13.3	1	0.821*	18.2	16.9^c^	12.8^d^
Mother	88.0	88.2	86.7	1.154 (0.335, 3.978)		81.8	83.1^c^	87.2^d^
Residence (%)								
Rural	41.3	38.2	42.7	1	0.663*	–	–	–
Urban	68.7	61.8	57.3	1.202 (0.525, 2.755)		–	–	–
Graffar class (%)								
I	8.3	11.8	6.7	–	0.514**	–	–	–
II	25.7	23.5	26.7					
III	56.0	50.0	58.6					
IV	10.0	14.7	8.0					
V	0.0	0.0	0.0					

* Calculated using Chi square test

** Calculated using Fisher`s test

^a^Binomial test to test against to target cohort proportion, p = 0.421

^b^Binomial test to test against to target cohort proportion, p = 0.418

^c^Binomial test to test against to target cohort proportion, p = 0.319

^d^Binomial test to test against to target cohort proportion, p = 0.091

### Self-reported rates of adverse reaction to foods

**Fig. 1:**
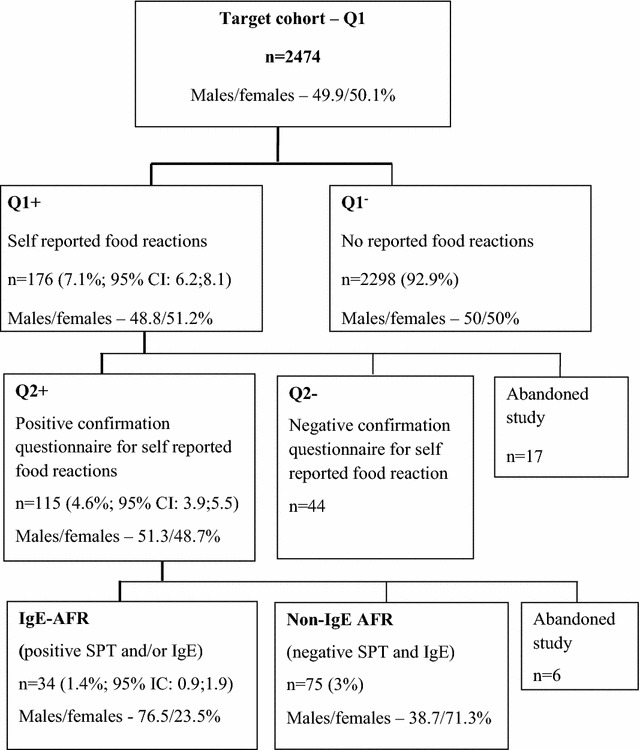
From self-reported adverse food reactions to confirmed sensitization—flow chart of the study process

In Q1 questionnaire, 176 reported adverse reactions to at least one food (7.1%)—Q1^+^ Group; mean age of 7.1 years; 48.8% males (Fig. 1).

Of these 176 children, 17 declined to continue the study (9.7% drop-out rate), since their parents did not wish to bring their children to the hospital for further studies. Thus, 159 children with filled in longer questionnaires (Q2), 115 reported an adverse reaction to food (4.6% in relation to the number of Q1 questionnaires)—Q2^+^ Group; mean age of 7.0 years; 51.3% males) (Fig. 1).

Both Q1 and Q2 were filled in by the parents; mothers filled in 81.8% of Q1 questionnaires and 83.1% of Q2 questionnaires.

### Atopy and prevalence of sensitisation to foods

Skin tests and determination of serum food allergen-specific IgE were carried out in all children with a positive Q2 questionnaire, with the exception of six, who declined to carry on in the study. In addition, the presence of atopy (using Phadiatop) was also studied in these 109 children. Atopy was present in 52.4% of these children. Thirty-four of these children had positive skin tests and/or allergen-specific IgE to at least one food, suggesting a prevalence of probable IgE-associated AFR of 1.4% in the target population; IC 95% 0.9–1.9, (IgE-AFR group). Negative skin test and allergen-specific IgE results were observed in 75 children (non-IgE-AFR group) (Fig. 1). The mean age was not significantly different between the IgE-AFR and non-IgE-AFR groups (6.65 ± 1.71 vs 7.16 ± 1.91 years, respectively),but the male/female ratio was significantly higher in the IgE-AFR group than in the non-IgE-AFR group (26/8 vs 29/46, respectively; p < 0.001; Chi square Test). In addition, atopy was significantly higher in the IgE-AFR than in the non-IgE-AFR group (Table 1).

### Foods implicated in adverse reactions

In the 115 Q2^+^ questionnaires, 259 foods were identified as suspect triggers. The most frequently implicated food groups were fresh fruits (83; strawberry, followed by kiwi fruit, orange and peach), egg (27) and fish (26) (Table 2). Of these 115 children, six abandoned the study (drop-out rate of 5.1%), for various reasons, including not wanting to subject their children to further tests namely because they already had a previous diagnosis or their children were successfully avoiding the suspect foods. In the 109 children who completed the full study, a total of 237 adverse food reactions were identified to various foods and analysed (Table 3). It should also be noted that, of these 109 children, 58% were sensitized to any aeroallergen and 44.7% were sensitized to pollens (mostly grass pollen—40%, and tree pollens, mostly olive tree—33%). Of the 78 analyzed food reactions to fresh fruits, 28 were IgE-associated reactions and 50 had negative fruit-specific IgE levels and SPT. Of the 28 cases of IgE-associated reactions, 24 had elevated fruit-specific IgE levels and 12 had positive SPT. Finally, all children sensitized to strawberry, pear and peach were sensitized to grass pollens.

**Table 2:** Foods implicated in adverse food reactions in Q2, in IgE-associated AFR and in non-IgE-associated AFR

Implicated foods	Q2^+^	Non-IgE AFR (SPT−/IgE−)	IgE-AFR (SPT/IgE+)	% confirmation of IgE-associated mechanisms^a^
Total children	*115*	*75*	*34*	
Total foods	*259*	*149*	*88*	*37.1*
Fresh fruits	83	50	28	35.9
Egg (white and/or yolk)^b^	27	11	14	56.0
Crustaceans/mollusks	23	17	4	19.1
Leguminous	19	8	6	42.9
Milk	17	11	5	31.3
Fish	26	8	18	69.2
Other vegetables^c^	16	12	2	14.3
Spices	20	17	1	5.6
Meat	4	3	1	25.0
Cereals	12	6	6	50.0
Nuts	10	4	3	42.9
Other^d^	2	2	0	0.0

^a^ % confirmation = IgE-associated cases/(IgE-associated + non IgE-associated) × 100

^b^As described in text

^c^Peppers, onion, tomato, spinach, celery, cress, cabbage

^d^Honey

**Table 3:** Clinical manifestations of adverse food reactions in 109 children who fully completed the study

AFR	IgE-AFR	%	Non-IgE-AFR	%	OR (95% CI)^a^	*p* value*
(n = 237)	(n = 88)		(n = 149)			
Immediate						
No	15	17.0	81	54.4	1	<0.001
Yes	73	83.0	68	45.6	5.797 (3.050–11.020)	
Poly-symptomatic						
No	51	58.0	122	81.9	1	<0.001
Yes	37	42.0	27	18.1	3.278 (1.810–5.938)	
Urticaria/angioedema						
No	21	23.9	54	36.2	1	0.048
Yes	67	76.1	95	63.8	1.814 (1.002–3.282)	
OAS						
No	50	56.8	116	77.8	1	0.001
Yes	38	43.2	33	22.2	2.672 (1.507–4.734)	
Gastrointestinal						
No	61	69.3	98	65.8	1	0.575
Yes	27	30.7	51	34.2	0.851 (0.483–1.497)	
Respiratory						
No	53	60.2	139	93.3	1	<0.001
Yes	35	39.8	10	6.7	9.179 (4.247–19.839)	
Other^b^						
No	67	76.1	125	83.9	1	0.141
Yes	21	23.9	24	16.1	1.632 (0.847–3.148	
Anaphylaxis						
No	56	63.6	143	96.0	1	<0.001
Yes	32	36.4	6	4.0	13.619 (5.400–34.348)	

* Calculated using Chi square test

^a^ *OR odds ratio*: For each categorical variable, the “normal” situation was defined as the reference category and odds were estimated for the others categories against the reference one

^b^Atopic eczema aggravated, headache, change in urine

In the 34 children of the IgE-AFR group, upon test-based confirmation, 88 foods were identified as triggers, with a mean of 2.6 foods per child (Tables 2, 3). Fifty per cent of these children were sensitized to two or more foods. The most frequent food groups in the context of IgE-associated sensitization were fresh fruits (kiwi fruit, peach and strawberry), fish and egg. In the 75 children of the non-IgE-AFR group, 149 foods were implicated in the reactions, most frequently fresh fruits (50), spices (17), and shellfish (17). Of all cases of self-reported adverse food reactions, IgE-associated sensitization was confirmed more frequently to fish (69%) and egg (56%). Of the 14 children who were sensitized to egg, four were exclusively sensitized to egg white and the remainder were sensitized to both white and yolk. Anaphylaxis was moderate in two cases of total egg (white and yolk) sensitization, and mild in two cases of egg white sensitivity and in four cases of total egg sensitivity.

### Clinical features of adverse food reactions

Ingestion was the trigger for all reported reactions. However, cutaneous contact with foods was significantly more frequently identified as a trigger for reactions in the IgE-AFR group than in the non-IgE-AFR group (27/88 vs 2/149, respectively; p < 0.0001—Fisher’s exact test), and this was essentially associated with fish.

IgE-AFR were also significantly more frequently associated with earlier clinical manifestations upon ingestion of foods and with poly-symptomatic manifestations than non-IgE-associated reactions (Table 3).

In the IgE-AFR group, the most frequent clinical manifestation were mucocutaneous and respiratory. In contrast, in the non-IgE-AFR group, mucocutaneous manifestations and gastrointestinal symptoms were very frequently reported and most cases were mono-symptomatic. Mucocutaneous, respiratory and anaphylactic manifestations were significantly more frequent in the IgE-AFR group than in the non-IgE-AFR group (Table 3).

The first adverse reaction to food occurred at a significantly earlier age in children of the IgE-AFR group when compared with the non-IgE-AFR group (1.9 versus 3.0 years of age, respectively; p < 0.001; Student’s *t* test).

Reactions were similarly reproducible upon re-ingestion of foods in both IgE-AFR and non-IgE-AFR groups, with consistent reactions developing in a high percentage of cases (77.3 and 74.5%, respectively).

### In vivo and in vitro studies of IgE-mediated reactions to food

Of the 88 foods tested, elevated levels of allergen-specific IgE were detected in 78 cases and positive SPT and/or SPPT were positive in 47 cases (Table 4). All foods that were positive in SPPT were also positive in SPT. IgE levels were more frequently elevated than were SPT positive, for most food groups.

**Table 4:** In vitro and in vivo studies with foods implicated in IgE-associated AFR

	Specific IgE (kUA/L)					SPT		IgE-associated foods (n)
	Positive (n)	Geometric mean	s.e.m.	Min	Max	Positive (n)	Weal size mean ± SD (mm)	
Total number of cases	78	2.83	1.69	0.40	81.80	47	5.32 ± 2.14	88
Fresh fruits	24	3.02	3.49	1.00	81.80	12	4.67 ± 1.27	28
Fish	17	3.54	1.45	0.76	21.50	12	6.21 ± 2.37	18
Eggs	12	2.77	2.47	0.39	29.00	8	5.06 ± 1.70	14
Legumes	5	4.72	7.11	1.58	38.60	4	7.63 ± 4.03	6
Cereals	6	1.49	8.10	0.44	49.40	1	3	6
Milk	5	1.73	1.15	0.37	6.59	1	4	5
Shellfish	4	2.73	7.42	0.36	31.30	3	3.83 ± 1.04	4
Nuts	3	2.35	25.86	0.40	78.00	2	5.75 ± 1.06	3
Other (vegetables, pork, spices)	2	1.91	1.83	0.82	4.47	4	4.00 ± 0.71	4

*s.e.m.* Standard error of the mean, *SD* standard deviation

### Food type-linked clinical features of IgE-associated reactions to food

Some significant differences were observed between the three most frequently reported foods, in terms of food-induced clinical manifestations in IgE-associated cases (Table 5).

**Table 5:** Food type-linked clinical features of IgE-associated reactions to food

Foods	Fresh fruits (n = 28)	%	Fish (n = 18)	%	Egg (n = 14)	%	*p* value*
Immediate reaction	25	89.2	18	100	10	71.4	0.035
Urticaria/angioedema	16	57.1	18	100	11	71.4	0.002
OAS	18	64.3	10	55.6	3	21.4	0.030
Respiratory	6	21.4	10	55.6	6	42.9	0.055
Gastrointestinal	6	21.4	6	33.3	8	57.1	0.069
Anaphylaxis	4	14.3	9	50.0	8	57.1	0.006

* Chi square test or Fisher’s exact Test as appropriate

All reactions to fish were immediate and most involved cutaneous and respiratory manifestations. In contrast, fresh fruits were most commonly associated with oral allergy syndrome (OAS) whereas egg related reactions were less frequently immediate and most commonly involved gastrointestinal or anaphylactic symptoms.

When clinical manifestations were broken down according to foods, urticarial/angioedema episodes were most frequently reported with fish. OAS was essentially observed with fresh fruits (64% of fruit-sensitised children reported OAS; all of these children were also sensitized to pollens—mainly grass pollens, with or without sensitization to tree pollens) and fish, respiratory symptoms were most commonly associated with fish and egg, and gastro-intestinal symptoms and anaphylaxis were most frequently reported upon ingestion of egg and were much less frequent with fresh fruits. Since fresh fruits were an important trigger of food allergies, we further characterized the specific clinical features of adverse food reactions to most frequently associated fresh fruits. In this context, all of the most frequently associated foods (kiwi, peach and strawberry) were most commonly associated with OAS (75% of all cases of IgE-associated kiwi or strawberry fruit allergy; 80% of all IgE-associated peach allergy cases). Kiwi fruit was the only one which was associated with gastrointestinal manifestations (33% of all cases of IgE-associated kiwi fruit allergy), whereas respiratory symptoms and anaphylactic episodes were only induced by kiwi fruit and peach.

## Discussion

This report is the first population-based study of the prevalence of adverse food reactions in children in Portugal. We obtained a satisfactory reply rate (61.2%) to the initial questionnaire from the parents of children attending public schools and pre-schools in the centre of Portugal. Prevalence of self-reported adverse food reactions (perceived food allergy) was 4.6%, and the prevalence of probable IgE-associated food allergy (IgE-AFR) was 1.4%.

In Portugal, there is only one previous study of the prevalence of food allergies in children but which was carried out in an allergy clinic [11]. However, in contrast to our study, that report was not carried out in the general population and include children and adolescents up to 18 years of age. Overall, in our study, the prevalence of in vivo (SPT) and in vitro (food-specific IgE levels) test-confirmed, probable IgE-associated food allergy was 1.4%. This is close to the prevalence values found in other studies that included a similar approach [16–18]. Since we did not perform oral provocation tests with suspect foods, our point prevalence values are higher than those obtained in studies using such tests [18–23]. This limitation may lead us to overestimate the prevalence of confirmed food allergy. Although we proposed performing provocation tests in children with positive questionnaires, most parents did not consent to the test being performed because they were satisfied with a clinical history-concordant diagnosis of probable food allergy.

We found a prevalence of self-reported adverse food reactions of 7.1%, when based upon our initial, screening questionnaire (Q1), and of 4.6%, when based upon a more thorough, confirmatory questionnaire, applied by the researchers (Q2). Such a difference in self-reported AFR values was also found in a French study, since an initial questionnaire given to parents of 2.5–14 year old children showed a prevalence of 6.7% but a subsequent, confirmatory questionnaire only found a prevalence of 4.7% [24]. Remembering previous episodes of food-associated symptoms may depend upon how recent and how severe the reaction was, whether the parents witnessed it or not or whether there have been more than one episode, possibly leading to memory bias, and a careful interview may reduce such bias. An even lower prevalence value was found when we consider confirmed IgE-associated AFR in Q1-positive children in our study—1.4%, corresponding to 19.3% of all Q1^+^ cases, a value which is similar to that reported in other studies [5, 21], and which supports the notion that adverse food reactions are over-reported by parents, as compared with results from confirmatory tests [5, 18, 20, 21, 23], often leading to unnecessary eviction diets [25–27].

Fresh fruits were, in all phases of our study, the most frequently implicated food group. Fish and egg were also high-risk foods where the suspicion of food allergy was frequently confirmed. Curiously, in the non-IgE AFR group, spices and shellfish were frequently reported. Our results are different from those more frequently reported in children in other countries, in which the most prevalent foods have been cow’s milk, peanut, eggs, or wheat [18, 24, 28] although a German study in children and adolescents also found fruits as the most frequently reported and confirmed cause of food-induced symptoms [19]. Previous Portuguese reports also showed a high relevance of fresh fruits in AFR in adults [10, 29] and in children with an age range similar to that in our study [11], and similar results were observed in Spanish children [30]. Since the diet followed by children in our study is similar to that in other regions of Portugal, the high prevalence of probable allergy to fresh fruits and fish may be due to the mediterranean type of diet of the population.

Some cases of cow’s milk allergy were those with the earliest onset. Interestingly, we found that the first episode of an adverse food reaction occurred significantly earlier in the IgE-AFR than in the non-IgE-AFR group (1.9 vs 3 years of age, respectively). As far as we know, this is the first report of such finding in the literature. Although this may be due to differences in the mechanisms of the underlying reaction, or the foods involved, it may also be due to the fact that IgE-AFR tend to be more severe than non-IgE-AFR and, therefore, a first episode of IgE-AFR may be more easily remembered (memory bias). Nevertheless, we believe that our results may indeed reflect a true difference in the age of onset since our questionnaire aimed at confirming such data and almost all parents gave precise records of the first episode. In any case, the mean age at which the first episode of non-IgE-AFR occurred is similar to that found in other questionnaire-based studies elsewhere [24, 27].

As shown in a Spanish study [30], our cases of IgE-AFR were more frequently polysymptomatic and of early onset. In addition, as demonstrated in other reports in children [23, 24, 28], our study showed that the most frequent clinical manifestations were mucocutaneous. Respiratory symptoms and anaphylaxis were significantly more frequent in the case of IgE-AFR than in non-IgE-AFR (39.8 vs 6.7 and 36.4 vs 4.0, respectively). The prevalence of anaphylaxis (36.4%) was much higher than that observed in other studies, between 0 and 15.6% [21, 24, 28]. These differences may depend upon the profile of sensitising foods, as well as whether reactions are IgE-mediated or not, although they may also possibly be due to different age groups under study or variations in the diagnostic methodology. Interestingly, we detected significant differences in terms of clinical symptoms which were reported with the three food types that were most commonly associated with adverse food reactions (fresh fruits, fish and egg). Very few studies have performed this type of specific food-associated symptom analysis. However, a French study, carried out in schoolchildren of a similar age range [24] also showed that egg ingestion was most frequently associated with cutaneous symptoms, followed by gastrointestinal symptoms. Nevertheless, a further comparison cannot be made with our study since these authors reported other food types.

Our study has some limitations. First of all, we must also fully accept that since we did not perform oral provocation tests with suspect foods, this may lead us to overestimate the prevalence of confirmed food allergy. However, as happens with other similar studies worldwide, our study nevertheless yields very important data regarding IgE-associated and non-IgE-associated AFR. Furthermore, the interpretation of cut-off values for positivity and future studies in children with IgE-associated AFR should address the possibility of defining positive and negative predictive values for a positive oral provocation test, although this depends upon each type of food. Further studies are warranted in Portugal.

Secondly, in terms of non-IgE-associated AFR, we cannot fully distinguish between situations that may involve intolerance to food, other forms of non-IgE-mediated food allergy and some paediatric gastroenterological situations. However, in our study, children with non-IgE associated reactions were fully studied in gastroenterological terms and we believe that we were able to exclude most paediatric gastroenterological and metabolic situations.

Thirdly, although our results involve a broad region comprising most of central Portugal, caution should be applied in terms of generalization of results. However, this applies to most international studies since most of them were carried out in single cities.

Finally, it is possible, as described in other studies, that some of the responses given by the parents are subject to memory bias. Still, we were very rigorous in terms of confirmation of all reported data, by searching all previous clinical records, besides obtaining information from both parents and, when justified, from other relatives.

## Conclusions

In conclusion, this first population-based study showed that the prevalence of probable food allergies in children from central Portugal was low and that parents tend to over-report its frequency. Most frequently implicated foods were fresh fruits and fish. Immediate type, polysymptomatic, and more severe reactions may commence at an earlier age and be more frequent in IgE-associated than in non-IgE-associated reactions. Our study has contributed to the characterization of adverse food reactions in Portuguese children.

## Additional file

**Additional file 1.** Questionnaire for screening adverse reactions to foods.

## Abbreviations

AFR: adverse food reaction
CI: confidence interval
IgE: immunoglobulin E
IgE-AFR: IgE-associated adverse food reaction
Non-IgE-AFR: non-IgE associated adverse food reaction
OAS: oral allergy syndrome
OR: odds ratio
Q1: questionnaire number 1 (initial, screening food questionnaire)
Q1^+^: questionnaire 1 with positive responses to AFR-related questions
Q1^−^: questionnaire 1 with negative responses to AFR-related questions
Q2: questionnaire number 2 (expanded, confirmatory food questionnaire)
Q2^+^: questionnaire 2 with positive responses to AFR-related questions
Q2^−^: questionnaire 2 with negative responses to AFR-related questions
s.e.m.: standard error of the mean
SPPT: skin prick by prick test
SPT: skin prick test
