# Supplementary material for: Prevalence and clinical features of adverse food reactions in Portuguese children
Source: Allergy Asthma Clin Immunol. 2017 Sep 6;13:40. doi: 10.1186/s13223-017-0212-y (PMC5585952; doi:10.1186/s13223-017-0212-y)
Supplement: Supplementary file 1 — Additional file 1. Questionnaire for screening adverse reactions to foods. [file 13223_2017_212_MOESM1_ESM.pdf]

QUESTIONNAIRE FOR SCREENING ADVERSE REACTIONS TO FOODS

Date:____/____/_____
Name:_______________________________
Address:____
Date of Birth : ____/____/____ Age: ______ years
Gender: □ Male □ Female
School: ____
Interviewee: □ mother □ father □ other

A. Does your child have any health problem or reaction with any food or drink?

□ Yes □ No □ Does not know

B. Which food or drink triggers a reaction?

1. Milk……………□ Yes □ No □ Does not know
2. Egg…………….□ Yes □ No □ Does not know
3. Wheat………….□ Yes □ No □ Does not know
4. Fish…………….□ Yes □ No □ Does not know
5. Soya…………….□ Yes □ No □ Does not know
6. Peanut………….□ Yes □ No □ Does not know
7. Shrimp………….□ Yes □ No □ Does not know
8. Other Shellfish…□ Yes □ No □ Does not know
9. Pork…………….□ Yes □ No □ Does not know
10. Fresh fruit……..□ Yes, Which?____________________□ No □ Does not know
11. Legumes……...□ Yes, Which?____________________□ No □ Does not know
12. Other …………□ Yes, Which?____________________□ No □ Does not know

(from C to P ask about each food, if there is more than one suspected food)

C. When your child had the reaction, was that the first time that he/she ate (or drank) that food? Yes/no/doesn not know. How old was your child when that reaction took place?

Age / Suspected food	1st ingestion	1st reaction	Does not know

D. How long after having eaten the food did the reaction occur? (Await spontaneous response and only read the options subsequently)

**Suspected food**	**Up to 2 hours / (min)**	**More than 2 hours/ (H)**	**Biphasic**	**Does not know**

E. What type of reaction did your child have after having eaten / drunk that food/drink?
(Await spontaneous response and only read the options subsequently)

**Suspected food** / **Symptoms**			
Cough			
Sneezing bout			
Nasal congestion			
Shortness of breath			
Itchy mouth or throat			
Swelling of lips, mouth or throat			
Itchy eyes			
Swelling of eyelids			
Swelling of face, ears, hands or feet			
Itchy skin			
Red or hot skin			
Skin rash (macules and papules)			
Nausea or vomiting			
Diarrhoea			
Abdominal pain or cramps			
Abdominal bloating			
Blood in stools			
Constipation			
Headaches			
Dizziness or fainting			

Other? Which type? Sweating, pallour, cyanosis, syncope, palpitations, low blood pressure			

F. How was the reaction triggered by the food/drink?

Suspected food	Direct Contact (mucosal)	Inhalation	Ingestion	Does not know

G. Was your child taken to hospital when he/she had the reaction to food / drink?

Suspected food	No	Yes, to casualty department; same day	Yes, on a different day	Does not know

H. Did your child have to be given any medication when he/she had the reaction?

Suspected food	No	Adrenaline	Corticosteroid	Bronchodilatador	Antihistamine	Does not know

I. After the first reaction, did your child evere eat that food/drink again? Did he/she have another reaction? What type of reaction did he/she have?

Suspected food	Yes			No	Does not know
	The same	Other (What type?)	None		

(Same list of symptoms as for question E)

J. In total, how many episodes of adverse reactions to the same food did your child have?

Suspected food	1	2-5	>5	Does not know

K. If your child ate the food more than once, have reactions to it changed in severity over time, to the same food?

Suspected food	All reactions of same intensity	First reaction was the most severe one	Reactions have become more severe	Does not know

L. If your child smells that food or it touches his/her skin, does he/she have any reaction?

(shortness of breath, nasal congestion, erythema, itch, urticarial rash, other)

Suspected food	Contact	Inhalation	Both	No reaction	Does not know

M. Were factors such as physical exercise, ingestion of medication or any other, associated with the reactions to the foods?

Suspected food	Exercise	Drug (name?)	Other	No reaction	Does not know

N. How long ago did the last reaction take place?

Suspected food	< 1 Month ago	1 month – 1 year ago	1-5 years ago	> 5 years ago	Does not know

O. Did your child ever have itchy, swollen or tingling lips, mouth or throat after having eaten any other food? (open question, followed by asking about any fresh fruit or legumes)

Suspected food	Yes (describe symptoms)	No	Does not know

P. Does your child have any other allergies?

	Yes		No	Does not know
Asthma				
Nasal allergies / rhinitis				
Cutaneous / atopic dermatitis				
Eye allergies /allergic conjunctivitis				
Other		Describe:		

Q. Does anyone in the child's family have any allergies?

	Mother	Father	Brother / Sister
**Food allergies**			
**Asthma**			
**Allergic rhinitis**			
**Allergic Conjunctivitis**			
**Atopic Dermatitis**			
